# Effect of Constant Temperatures on *Culicoides sonorensis* Midge Physiology and Vesicular Stomatitis Virus Infection

**DOI:** 10.3390/insects13040372

**Published:** 2022-04-09

**Authors:** Paula Rozo-Lopez, Yoonseong Park, Barbara S. Drolet

**Affiliations:** 1 Department of Entomology, Kansas State University, Manhattan, KS 66506, USA; paularozo@ksu.edu (P.R.-L.); ypark@ksu.edu (Y.P.); 2 Arthropod-Borne Animal Diseases Research Unit, Center for Grain and Animal Health Research, Agricultural Research Service, United States Department of Agriculture, Manhattan, KS 66502, USA

**Keywords:** *Culicoides* midges, vesicular stomatitis virus, constant temperature, thermal preference, vector competence

## Abstract

**Simple Summary:**

*Culicoides* biting midges are nuisance pests of livestock and well-known vectors of veterinary arboviruses, such as vesicular stomatitis virus (VSV). Female midges ingest viruses when feeding on blood to obtain protein for egg-laying. After ingesting a VSV-infected blood meal, the environmental temperature of the resting location mediates the rates at which blood is digested, eggs are laid, and virus particles are replicated inside the midge. VSV transmission will occur if the timing of virus amplification aligns with the next feeding–egg-laying cycle. We evaluated the impact of constant environmental temperatures on midge physiology (lifespan and reproduction), vector competence for VSV (infection and dissemination), and thermal resting preference. Our results indicate that after ingesting a blood meal, most midges prefer to rest in areas that fall within their preferred physiological range regardless of the temperatures at which they were being maintained. These preferred temperatures maximized their survival, the number of egg-laying cycles, and the likelihood of VSV transmission. Our temperature approach shows that in the *Culicoides*–VSV system, the preferred resting temperature selected by blood-fed midges is beneficial for both insect and virus transmission.

**Abstract:**

*Culicoides* midges play an important role in vesicular stomatitis virus (VSV) transmission to US livestock. After VSV-blood feeding, blood digestion followed by oviposition occurs while ingested virus particles replicate and disseminate to salivary glands for transmission during subsequent blood-feeding events. Changes to environmental temperature may alter the feeding–oviposition–refeeding cycles, midge survival, VSV infection, and overall vector capacity. However, the heterothermic midge may respond rapidly to environmental changes by adjusting their thermal behavior to resting in areas closer to their physiological range. Here we investigated the effects of four constant environmental temperatures (20, 25, 30, and 35 °C) on *C. sonorensis* survival, oviposition, and VSV infection, as well as resting thermal preferences after blood-feeding. We found that most midges preferred to rest in areas at 25–30 °C. These two constant temperatures (25 and 30 °C) allowed an intermediate fitness performance, with a 66% survival probability by day 10 and oviposition cycles occurring every 2–3 days. Additionally, VSV infection rates in bodies and heads with salivary glands were higher than in midges held at 20 °C and 35 °C. Our results provide insight into the implications of temperature on VSV–*Culicoides* interactions and confirm that the range of temperature preferred by midges can benefit both the vector and the arbovirus.

## 1. Introduction

*Culicoides* midges (Diptera: Ceratopogonidae) are well-known nuisance pests and arbovirus vectors with worldwide epidemiologic implications on a wide variety of agricultural host species [1,2,3]. *Culicoides sonorensis* is one of the most common midge species associated with livestock across the continental US [4] and are efficient biological vectors of the rhabdovirus, vesicular stomatitis virus (VSV), causing disease in cattle, horses, sheep, goats, llamas, alpacas, and swine [5,6,7,8,9,10,11,12]. The clinical resemblance of vesicular stomatitis with foot-and-mouth disease in cattle and swine leads to quarantines and trade embargoes, which, along with a reduction in animal production, generate significant economic losses in affected premises [13].

Among climatic conditions, temperature plays the most significant role in the ecology of the heterothermic *Culicoides* midges and the viruses they transmit, mainly by constraining midge seasonality, distribution, and abundance [1,14,15,16]. In the last 20 years of rising global temperatures, *Culicoides*-borne virus emergence and re-emergence have increased [14,17] due to the geographical expansion of vector species [18,19,20,21,22,23,24], the increase in their population sizes [25,26,27], and physiological alterations that may favor their susceptibility to viruses [16,28,29,30,31]. However, local midge populations may respond rapidly to environmental changes at the individual level by behaviorally thermoregulating and seeking out microclimates within their optimal physiological range [32].

Adult *Culicoides* females must ingest a blood meal to produce eggs [33]. Females from many *Culicoides* species blood-feed in swarms on a wide variety of vertebrate hosts but feed preferentially on domestic and wild ruminants and horses [34,35]. While taking small blood meals [36,37], the success of midges as vectors is primarily due to large population sizes that can be sustained with suitable climatic conditions [1]. Under standard laboratory conditions (25 °C and 70% RH), adult *C. sonorensis* females can complete three to four gonotrophic cycles (GC) within their three- to six-week lifetime [38]. However, lifespan and blood-feeding frequency are strongly linked to temperature-mediated metabolic rates [38,39,40,41]. Understanding how temperature impacts midge survivorship and the length of the GC allows us to calculate the vector abundance and feeding frequency, thereby estimating their vectorial capacity (Vc) [32].

The Vc is determined by the environmental, behavioral, and physiological factors that influence the association between vector, virus, and host [42]. This measure of transmission potential accounts for vector density, longevity, blood-feeding rates, and the extrinsic incubation period (EIP) [42,43]. *Culicoides* adult survival and the period between successive blood meals are the major determinants of the probability of arbovirus transmission; however, both parameters are impacted by temperature in diametric opposition [44]. Rising environmental temperatures increase the blood-feeding frequency by accelerating the egg development rate but shortening the midge lifespan [38,39,40]. *Culicoides* biting activity also positively correlates with an optimal temperature range, constrained by lower and upper thresholds at which feeding is suppressed [1]. Although there is variation in Vc between *Culicoides* species, common barriers or limitations in the expected number of bites per day, temporal peaks in *Culicoides* abundance, temperature-dependent vector mortality, and the time interval between feeding events could shift in response to climate change and associated habitat expansions [45].

In contrast, the EIP component of Vc is mainly determined by the time required for the vector to become infected and subsequently transmit the virus [3,42]. With VSV oral infection, the virus particles ingested with the bloodmeal must replicate in the midgut epithelium, escape the midgut, be released into the hemocoel, and subsequently infect a range of secondary target organs, including the salivary glands [11]. The progeny virions accumulate in the salivary gland lumen and are transmitted in saliva during subsequent blood-feeding [3,11]. Under standard laboratory conditions, VSV-disseminated infections have been reported as early as 3 days post-feeding [11], suggesting a 3-day EIP, which aligns well with the 3–4 day feeding–egg-laying cycle observed under those conditions. However, epidemiological models for many *Culicoides*-borne viruses indicate that current increasing temperatures may shorten the EIP in a non-linear fashion [46].

Optimal arbovirus transmission occurs when the timing of productive infection (virus–vector interactions) aligns with the timing of feeding–ovipositing–refeeding (vector–host interactions) [38,39,40]. Given the worldwide trend of increasing temperatures [47], it is expected that higher temperatures will impact most *Culicoides*–virus interactions by increasing the midge metabolic rates. Although not all processes may change in a linear manner, higher temperatures will likely increase individual mortality and biting rates and reduce the EIP [3,38,39,40,46]. Therefore, some suggest that the short-lived, yet highly competent *Culicoides* may become a minor vector species if this variation in environmental temperatures result in significant reductions in midge lifespan and/or a misalignment between the EIP and the blood-feeding frequency [46].

The influence of temperature on *Culicoides* adult biology has been previously explored in oocyte development [32], seasonal abundance [48], and flight activity [23,49], as well as some aspects of vector competence for orbiviruses [3,29,31,39,46,50,51,52]. However, studies addressing the temperature-mediated effects on VSV infection and transmission risk at the microclimate level are lacking. Given the current trajectory of rising global temperature and the possibility of rapid adaptation through behavioral modifications [32], here we explored *C. sonorensis* resting thermal preferences after engorgement with three sequential blood meals and investigated how four constant temperatures (20, 25, 30, and 35 °C) may influence *Culicoides-*VSV interactions.

## 2. Materials and Methods

### 2.1. Virus and Cells

Stock virus (VSV-NJ; 1982 bovine field isolate, USDA-APHIS, Ames, IA, USA) was grown in porcine epithelial cells (AG08113; Coriell Institute, Camden, NJ, USA) in Eagles Minimum Essential Medium (MEM) with Earle’s salts (Sigma, St. Louis, MO, USA) containing 2% FBS and 100 U penicillin/streptomycin sulfate at 37 °C with 5% CO_2_. Vero MARU cells (VM; Middle America Research Unit, Panama City, Panama) grown in 199E media containing 2% FBS, 100 μg/mL of streptomycin, 100 units/mL penicillin, and 0.25 μg/mL of amphotericin B at 37 °C with 5% CO_2_ were used for detecting the virus by cytopathic effect (CPE) and for titering the virus from midge samples, as described below.

### 2.2. Blood-Feeding and VSV Oral Infection

Colonized *C. sonorensis* adult midges (AK colony, USDA, Arthropod-Borne Animal Diseases Research Unit, Manhattan, KS, USA) were used for all experiments. Adult midges were maintained in environmental chambers with 70 ± 5% RH and a 13:11 light:dark cycle and offered 10% sucrose solution *ad libitum*.

**Figure 1:**
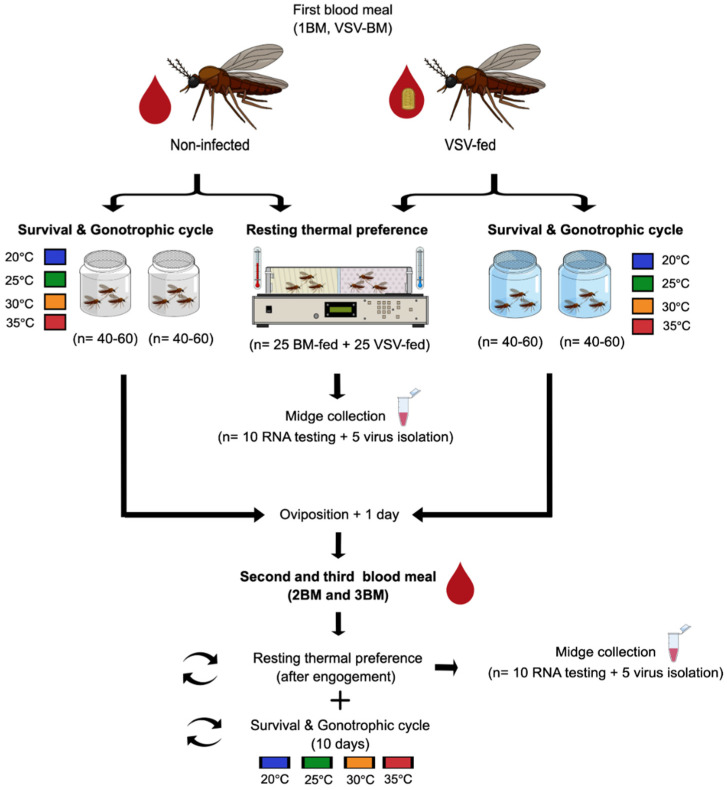
Experimental design to test constant temperature-mediated effects on post-engorgement resting temperature preference, midge survival, gonotrophic cycles, and VSV infection.

For the first blood meal (1BM), newly emerged midges (1–3 days post-emergence) were offered either an infectious VSV-spiked bloodmeal (VSV-BM) or a non-infectious blood meal (BM) (Figure 1). Blood meals consisted of a 1:1 mixture of defibrinated sheep blood (Lampire Biological Products, Pipersville, PA, USA) and a VSV suspension in MEM (8.6 Log_10_ PFU of VSV-NJ per meal; VSV-BM) or MEM alone (non-infectious meal; BM). Midges were allowed to feed for 60 min on a water-jacketed (37 °C) glass bell jar feeder through parafilm (MilliporeSigma, St. Louis, MO, USA). After each feeding event, midges were anesthetized with CO_2_, fully engorged blood-fed females were sorted from unfed and partially fed, and 40–60 fully engorged females were placed into individual cardboard cages (4 oz) with a small cup (20 mm diameter) containing a water-moistened pad and a filter paper disk for oviposition.

Two cages of fully engorged females from each infection (BM-fed controls and VSV-BM fed midges) were placed into a secondary container and held at constant temperatures of 20 °C ± 0.5, 25 °C ± 0.5, 30 °C ± 0.5, or 35 °C ± 0.5 for up to 10 days. One day after oviposition, at the end of the first and second gonotrophic cycles (1GC and 2GC), both BM-fed controls and VSV-BM fed midges were provided subsequent non-infectious bloodmeals (2BM and 3BM). As above, 40–60 fully engorged females were sorted into cardboard cages and held at the same temperature at which they started the experiment (Figure 1).

In addition, after every feeding event (1BM, 2BM, and 3BM) 25 CO_2_-anesthetized, fully engorged females from each group were placed in cardboard cages and immediately used for resting thermal preference assays (Section 2.4; Figure 1).

### 2.3. Constant Temperature-Mediated Effects on Survival and Gonotrophic Cycles

Two groups of 40–60 VSV-BM fed and BM-fed controls held at constant temperatures of 20, 25, 30, and 35 °C were visually inspected at 24 h intervals for signs of oviposition (presence of eggs laid on oviposition cup filter paper) and mortality (dead midges at the bottom of the cages) for up to 10 days after the initial meal (maximum survival day for all groups). As indicated above (Section 2.2), one day after oviposition at the end of each GC, midges were provided subsequent non-infectious bloodmeals (2BM and 3BM), and fully engorged females were kept at the same environmental temperature at which they started the experiment (Figure 1). The effects of environmental temperature on the length of the GC (days) and midge probability of survival (days) were evaluated in four independent replicates consisting of two cages from each infection group (*n* = 40–60 midges per cage) for each temperature tested (total *n* = 3520 midges, 64 cages).

### 2.4. Resting Thermal Preference after Engorgement

The resting temperature preference after engorgement was evaluated using a thermal gradient comprised of two AHP-1200CPV cold/hot plates and a TGB-5030 aluminum bar (ThermoElectric Cooling America Corporation, Chicago, IL, USA) with a clear polycarbonate lid to contain the midges. A center lengthwise divider was used to accommodate two simultaneous testing groups (control and experimental) and to maintain stable temperature and humidity conditions within the arena. The experimental arena (60 × 30 cm) ranged from 15 °C to 35 °C, increasing linearly along the surface. The temperatures of the lid (8 cm above the gradient surface) ranged from 22 °C to 27 °C in a more non-linear fashion. To maintain humidity conditions during the experiment, polyethylene-coated chromatography paper was taped over the aluminum bar (glossy side down) and lightly sprayed with distilled water at the beginning of the experiment. Prior to the start of an experiment, each zone’s temperature range was manually confirmed using 2–4 consecutive measurements with an infrared laser thermometer (Simzo, Fisher Scientific, Inc., Waltham, MA, USA). To have a visual representation of the temperature preference across the length of the arena, the paper surface was marked into temperature zones: 15–20, 20–25, 25–30, and 30–35 °C; plus, one buffer area at the cold end (14 ± 1 °C) and one at the hot end (34 ± 1 °C).

Within 10–15 min of every feeding event (sorting time), 25 CO_2_-anesthetized, fully engorged females were loaded through small holes in the polycarbonate cover onto the starting zone (22–23 °C) at the center of the arena. Midges were allowed 15 min to recover from the CO_2_ exposure for maximum responsiveness during the initial exploratory period. During the next 15 min, midge movement slowed and ceased. At the 30 min mark, a single photo was taken to depict the distribution of midges across the temperature zones. After each trial, the arena was flooded with CO_2_ and all midges were collected using a vacuum pooter for virus testing as described below (Section 2.5 and Section 2.6).

To restrict circadian rhythm effects on *Culicoides* behavior, all blood feedings and resting preference trials were conducted at approximately the same time of day. All behavioral assays were conducted by simultaneously testing 25 fully engorged VSV-fed females and 25 blood-fed controls immediately after engorgement with 1BM, 2BM, and 3BM (Section 2.2; Figure 1). Seven independent replicates were performed for resting temperature preference after the engorgement of newly emerged females with the first blood meal (1BM or VSV-BM). Four behavioral assays were conducted with midges held at each temperature (20, 25, 30, and 35 °C) after the ingestion of the second BM (2BM; at the end of 1GC) and third BM (3BM; at the end of 2GC). A digital image was used to record midge position and determine the distribution frequency at each thermal zone selected by infected and non-infected midges.

### 2.5. RNA Extraction and RT-qPCR for Detection of VSV

Ten midges at the end of the resting thermal preference assays (Figure 1) were sampled as heads (with salivary glands attached) and decapitated bodies. Individual bodies and heads were sorted in 300 µL of TRIzol (Invitrogen; Thermo Fisher Scientific, Inc., Waltham, MA, USA) and stored at −80 °C until further processing. Frozen TRIzol samples were thawed on ice and homogenized by high-speed shaking with a Bead Mill Homogenizer (Omni, Kennesaw, GA, USA) [53]. Total RNA was extracted using Trizol-BCP (1-bromo-3chloropropane; Life Technologies, Thermo Fisher Scientific, Inc., Waltham, MA, USA), and RNA extracts were analyzed using TaqMan Fast Virus 1-Step MasterMix (Applied Biosystems; Thermo Fisher Scientific, Inc., Waltham, MA, USA) in a reverse transcriptase quantitative PCR (RT-qPCR) assay to detect the L (polymerase) gene, as previously described [7]. Standard curves and the calculation of Cycle threshold (Ct) values were carried out with the 7500 Fast Dx software (Applied Biosystems; Thermo Fisher Scientific, Inc., Waltham, MA, USA). RT-qPCR reactions with Ct ≤ 36.5 were considered positive for VSV RNA [7,53]. To account for plate-to-plate inter-run variations, a standard positive control of known VSV ssRNA concentration was used in every RT-qPCR run. Ct values plotted against the log_10_ of known VSV genome ssRNA ng concentrations with linear regression (y= −3.30578x + 11.02683) allowed the determination of viral genomic equivalents per midge [7]. Infection rates were calculated by dividing the number of VSV-RNA positive bodies by the total number assayed by RT-qPCR. Dissemination rates were calculated as the number of VSV-RNA positive heads divided by the number of VSV-RNA positive bodies.

### 2.6. Virus Isolation

Five individual females sampled at the end of the resting thermal preference assays (Figure 1) were collected in 500 µL of antibiotic medium [53] for virus isolation from whole bodies and stored at −80 °C until further processing. Frozen midges were thawed on ice and individually homogenized and centrifuged to pellet debris [53]. Whole-body homogenates (200 μL) were plated over a monolayer of VM cells with 85–90% confluency. Plates were incubated for up to six days. The observation of CPE after one or two passages was used to indicate an infectious virus within that sample [7]. If the homogenate was CPE+ in the first passage, virus titers were determined by a standard plaque assay using 200 μL of the remaining original, non-passaged homogenate. For homogenates that showed CPE after a second passage, VSV was confirmed in randomly selected wells by RT-qPCR, but no attempts to titer the original homogenate were made. The infectious virus-positive rate was calculated as the number of CPE+ whole-body homogenates divided by the number of midges assayed.

### 2.7. Statistical Analysis

Data were pooled from the independent replicates of each experiment and tested for normality (Kolmogorov–Smirnov test). For variables following a normal distribution, analysis of variance (ANOVA) with multiple comparisons (Tukey’s test) was used to compare the significance of the oviposition timing, resting thermal preferences, and Ct value differences of bodies. Non-parametric tests (Kruskal–Wallis with Dunn’s correction for multiple comparisons) were used to evaluate the significance of Ct value differences of heads (with glands) and the proportion of infected heads, bodies, and whole bodies. Kaplan–Meier curves and Mantel–Cox log-rank tests were used to evaluate survival and mortality rates. GraphPad Prism version 9 (GraphPad Software Inc., San Diego, CA, USA) was used for statistical analysis and the creation of graphs.

## 3. Results

### 3.1. Constant Temperature-Mediated Effects on Survival and Gonotrophic Cycles

**Figure 2:**
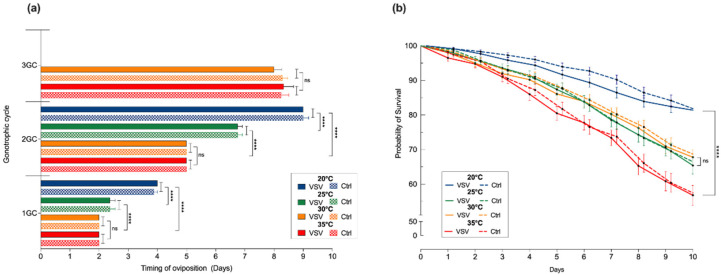
Constant temperature-mediated effects on (**a**) the gonotrophic cycle (GC) and (**b**) the survival of VSV-fed and non-infected control midges held at constant temperatures of 20 °C (blue), 25 °C (green), 30 °C (orange), and 35 °C (red). Two-way ANOVA with multiple comparisons was used to determine statistical significance in the timing of oviposition, as indicated (*p* > 0.05, ns, not significant; **** *p* ≤ 0.0001). Survival curves were calculated using the Kaplan–Meier method. Error bars represent the standard error of the mean (SEM).

Overall, the length of each GC (Figure 2a; Table S1) was affected by temperature but not infection status. Lower temperatures correlated with longer cycles, with significant differences between midges held at cold (20 °C), mild (25 °C), and hot (30 and 35 °C) temperatures. The average GC length was 4.5 days at 20 °C, 3.4 days at 25 °C, and 2.6 days at 30 and 35 °C, with the 1GC occurring faster (by one day less) than the two subsequent GCs in all temperature groups. Only midges held at higher temperatures (30 and 35 °C) were able to complete the 3GC within 10 days after ingesting their first blood meal. Likewise, the survival probability (Figure 2b; Table S2) was affected by temperature (Mantel-Cox model, *p* ≤ 0.0001) but not infection status. Lower temperatures correlated with higher survivability. For both VSV-fed and non-infected control midges, the day 10 survival probability was significantly different between midges held at 20 °C and 35 °C (81% and 57%, respectively, *p* ≤ 0.0001). There was no significant difference in the day 10 survival probability between midges held at 25 °C and 30 °C (65.7% and 68%, respectively, *p* = 0.8).

### 3.2. Constant Temperature-Mediated Effects on VSV Infection

**Figure 3:**
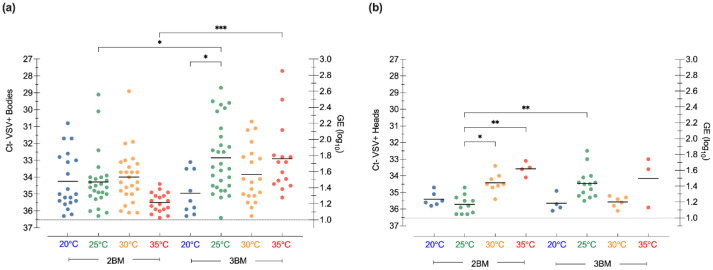
Constant temperature-mediated effects on VSV in individual (**a**) bodies and (**b**) heads with salivary glands, as detected by RT-qPCR of VSV-infected *Culicoides* midges after a second (2BM) and third (3BM) blood meal while being held at constant temperatures of 20 °C (blue), 25 °C (green), 30 °C (orange), and 35 °C (red). RT-qPCR cycle threshold (Ct; left Y-axis) and viral genome equivalents (GE; right Y-axis), as indicated. One-way ANOVA (bodies) and the Kruskal–Wallis test (heads) with multiple comparisons were used to determine statistical significance, as indicated (* *p* ≤ 0.05; ** *p* < 0.01; *** *p* < 0.001).

**Figure 4:**
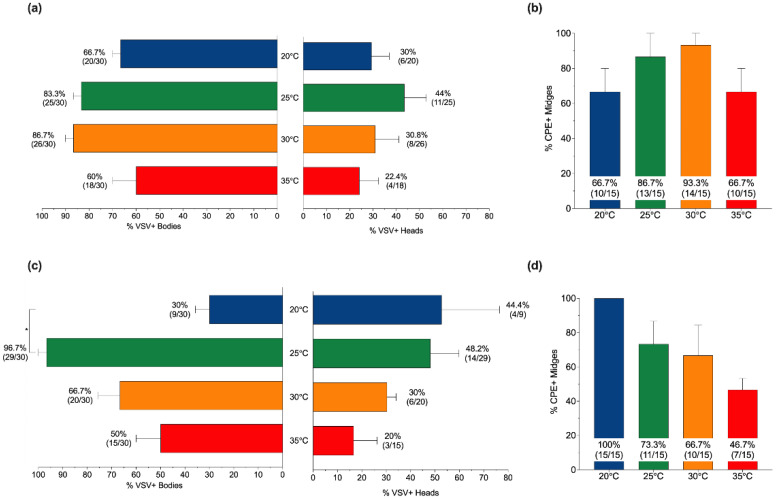
Constant temperature-mediated effects on infection rates of *Culicoides* midges orally infected and provided (**a**,**b**) a second (2BM) and (**c**,**d**) a third (3BM) blood meal while being held at constant temperatures of 20 °C (blue), 25 °C (green), 30 °C (orange), and 35 °C (red). (**a**,**c**) Infection rates of bodies and heads as detected by RT-qPCR and (**b**,**d**) infectious virus in whole bodies as detected by cytopathic effect (CPE) screening after one or two passages on Vero cells. The Kruskal–Wallis test with multiple comparisons was used to determine statistical significance (* *p*≤ 0.05). Error bars represent the standard error of the mean (SEM).

Following initial infection with VSV-BM, bodies from midges held at 20, 25, and 30 °C exhibited similar viral loads at 2BM (end of 1GC), ranging from 1 to 2.6 log_10_ genome equivalents (Figure 3a). Although not statistically significant, lower viral loads (genomic equivalents, GE) were observed in midge bodies held at 35 °C at 2BM (1 to 1.5 log_10_ GE). The viral load increased from 2BM to 3BM for midges held at 25 or 35 °C (1.8 log_10_ GE, *p* = 0.8 and 1.75 log_10_ GE, *p* = 0.005, respectively), but not for midges held at 20 or 30 °C. Only a significant difference in infection rates (Figure 4a) was found between midges held at 20 and 25 °C (*p =* 0.01), which coincides with the overall lowest (20 °C) and highest (25 °C) RNA titers detected in bodies by RT-qPCR (Figure 3a).

Regarding dissemination rates, VSV RNA was detected in heads of midges held at all temperatures (Figure 3b). Although midges held at 35 °C had the highest GE detected (2.9 log_10_; Figure 3b), they showed the lowest number of VSV-positive heads (Figure 4b). There was no significant difference in the VSV RNA titers between feeding events in the heads of midges held at 20, 30, and 35 °C (*p* > 0.99, *p* =0.33, and *p* > 0.99, respectively). In midges held at 25 °C, the mean RNA titer in the heads significantly increased between meals, reaching its highest mean value at 3BM (1.45 log_10_ GE, *p* = 0.009; Figure 3b). There were no statistically significant effects of temperature on dissemination rates (Figure 4b) or the detection of infectious viruses in whole bodies (Figure 4c).

### 3.3. Resting Thermal Preference

**Figure 5:**
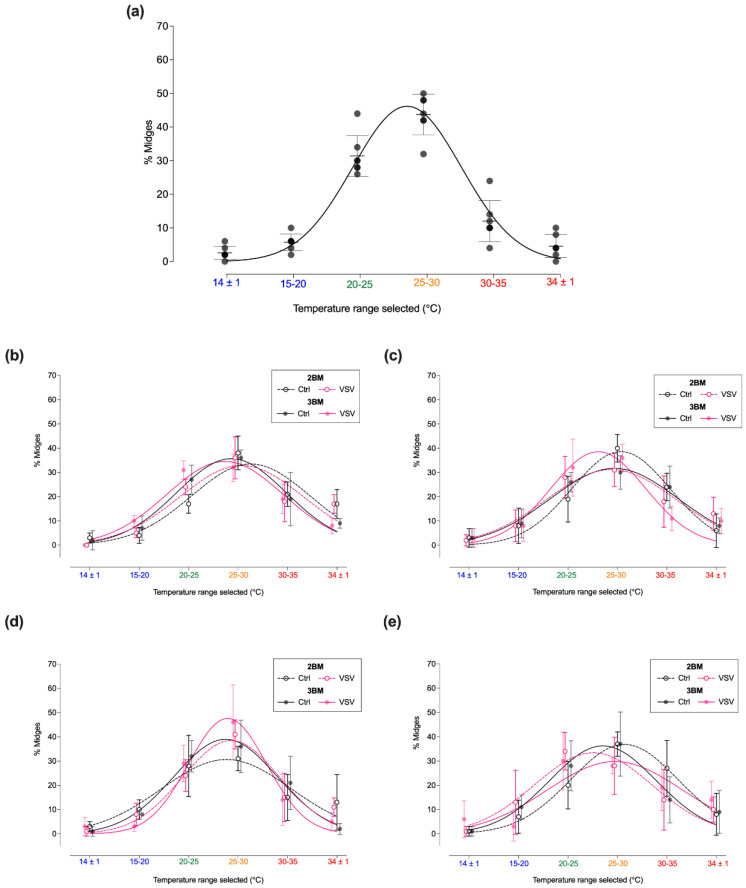
*Culicoides* resting temperature preference after engorgement. Mean final distribution of midges across the thermal gradient arena after engorgement with the (**a**) first blood meal (1BM and VSV-BM combined) and second (2BM) and third (3BM) blood meals in midges held at (**b**) 20 °C, (**c**) 25 °C, (**d**) 30 °C, and (**e**) 35 °C. Error bars represent the standard deviation (SD).

Immediately after engorgement with their first meal (VSV-BM or 1BM), there was no effect of the infectious status of the meal on the mean final distribution of newly emerged midges (1–3 days post-emergence, 25 °C) across the thermal gradient arena (*p* < 0.0001). Therefore, the data of both meal treatments were pooled to evaluate the resting temperature preference after the engorgement of midges with their first blood meal, prior to infection onset. Groups were separated as BM-fed or VSV-BM-fed for all subsequent meals. For the first meal, most midges selected the 25–30 °C thermal zone (43.7% ± 6; Figure 5a; Table S3). Likewise, most midges selected the 25–30 °C thermal zone after ingesting their 2BM and 3BM (Figure 5b–e; Table S3). Only VSV-infected midges held at 25 °C after 3BM (Figure 5c) and 35 °C (Figure 5e) either did not have a conspicuous option or selected the cooler 20–25 °C thermal zone.

A combined analysis of resting preferences across both subsequent meals (2BM, 3BM) indicated that midges prefer to rest in areas of the gradient above the starting zone (>22 °C) (three-way ANOVA; *p* < 0.0001). There was no effect of infectious status (*F*_1,24_= 0.21, *p* = 0.65) or the blood-feeding event (*F*_1,24_ = 0.02, *p* = 0.88) on the resting temperature preference of *C. sonorensis*, nor any interaction between the two (*F*_1,24_ = 0.34, *p* = 0.56).

## 4. Discussion

Environmental temperatures drive *Culicoides* seasonality and abundance [18,19,23,24,54,55], shaping vector-to-host ratios and influencing the probability that midge-borne viruses will become established following an introduction [1,16,56,57]. The most traditional equation to calculate vectorial capacity (Vc) accounts for the vector density, the number of blood meals taken, the vector survival rate, and the extrinsic incubation period (EIP) [16]. However, this model assumes that the EIP is proportional to the vector’s life expectancy, implying that the vector will survive the EIP [43,46]. To better understand how the resting temperature range selected by fully engorged *C. sonorensis* midges may mediate *Culicoides*–VSV interactions, we combined quantitative behavioral analyses with fitness-related traits and infection patterns at four constant thermal regimes to inform the potential Vc outcomes.

From the vector’s perspective, the most critical parameters for predicting vector-borne transmission are biting rates and survival [31,58]. Thus, we evaluated the length of the GC as an approximation of the feeding–oviposition–refeeding frequency during a 10-day lifespan. The results of this study are consistent with the expected effects of temperature changes on both fitness-related traits [32,38,39,40,41]. Midges showed the highest probability of survival (81%) and most extended GC length (4.5 days) at the lowest tested temperature (20 °C), and the lowest probability of survival (57%) and shortest GC lengths (2.6 days) at the highest tested temperature (35 °C). Interestingly, midges held at 30 °C were able to sustain the fastest oviposition–refeeding cycles (GC length of 2 days) while sustaining an optimal survival (67%).

Although most biological processes occur faster at higher temperatures, including the virogenesis rate during the EIP [52], there is a trade-off between viral transmission and vector life expectancy [43,46]. It has been suggested that the transmission potential of an infected vector is maximized at intermediate temperatures where the vector’s physiological performance aligns with the EIP and blood-feeding frequency [43,46]. Our study found that the highest infection rates and number of disseminated infections occurred in midges held at 25 °C and 30 °C. Moreover, our results indicate that VSV infection and dissemination rates are constrained at the lowest (20 °C) and highest (35 °C) temperatures evaluated. However, further experiments are needed to determine whether these temperatures impact infection and dissemination by acting directly on VSV replication or by acting indirectly on competence factors in the vector.

Lower temperatures slowed VSV replication rates, as seen by the lack of titer increase in bodies and heads between feeding events of midges held at 20 °C. Interestingly, we detected the highest percent of infectious virus (by CPE) in midges at this temperature. These results may suggest that slowing VSV replication rates at the lower temperature do not affect its infectivity. Although unknown for VSV, the threshold for infection and replication of most *Culicoides*-borne orbiviruses has been determined to lay within 11–15 °C [3,59,60]. It has been previously shown that *C. sonorensis* females can survive and complete an entire gonotrophic cycle (lasting 10–13 days) in temperatures as low as 13 °C [32]. Short-term exposure to low temperatures can stimulate cold-hardening responses in *C. sonorensis* adults, which allows a non-diapausing life stage to enhance its tolerance to subzero temperatures [61], and transient warmer periods in winter may be conducive to virus replication, leading to transmission when infected adults are able to survive, fly, and feed on hosts [49,59]. Anecdotal evidence during VSV outbreaks suggest that adult *Culicoides* may be the vector responsible for low rates of transmission seen during cool days (above freezing) in late fall and early spring. Therefore, it is critical to explore how changing climatic conditions may favor VSV overwintering in the adult stage in climates with moderately low winter temperatures.

In the context of *Culicoides*–VSV at 25 °C as the reference temperature (standard laboratory conditions), our results indicate that the Vc of a *Culicoides* population can be potentially maximized in a temperature range of 20–30 °C, and decreased at 35 °C. Although the Vc outcome under natural settings is difficult to predict due to fluctuating day/night temperatures, the complexity of the midge physiological responses, and the number of variables involved in its calculation, it is expected that rising global temperatures due to climate change will likely affect *C. sonorensis*–VSV dynamics. As seen with our results, constant temperatures near 30 °C will potentially provide an increased opportunity for virus transmission. A preferred resting temperature range of 25–30 °C may also favor the number of midge generations per year and, consequently, the number of adults and biting frequency, while maximizing the number of adults able to survive the VSV EIP. However, other variables such as daily and seasonal fluctuations, vegetation coverage, and air temperature can modulate the availability of ideal *Culicoides* macrohabitats [62,63]. Therefore, midge resting preferences to particular microhabitats can ensure that, regardless of the macroclimatic conditions outside the ideal, the actual temperatures experienced by a midge may still be within their optimal physiological range [28].

Interestingly, VSV-infected midges held at 35 °C either failed to respond or selected a cooler 20–25 °C thermal zone. This suggests that a combination of high temperatures and infection status might be shaping midge thermal behavior to prefer lower resting temperatures. In other vector–pathogen systems, thermal preference changes have been shown to limit the virulence of a pathogen or the influence of the infectious agent [64,65]. However, across all temperatures, our combined results indicated no effect of infectious status on resting temperature preference. It is important to note that our preference assays took place only for a short period after engorgement (30 min), and not 100% of the midges tested positive for VSV infection after the assays (30–96.7%). Thus, future thermotaxis analyses using microinjected midges (bypassing the midgut and ensuring positive infectious status) may be needed to fully determine whether the infectious status significantly influences thermal behaviors at the cost of potential effects from the injection.

In addition to temperature fluctuations in a natural context, other abiotic factors such as humidity and light must be considered. These parameters are rarely combined and studied in the laboratory under fluctuating conditions because the level of complexity could mask the effects of single variables. However, the preferred range of microclimatic conditions chosen by midges would allow for a better understanding of vector responses to climate change. In that sense, it is still necessary to evaluate midge thermotaxis in precisely controlled and ethologically relevant thermal gradients to determine if midges modulate their response to thermal cues on a daily cycle or if the temperature preference observed here would be more robust at specific times of the day [62,63].

*C. sonorensis* populations are widely distributed in the US, with a reported range spanning the western, south-central, mid-Atlantic, and southeastern states [4,66]. There are several ecological regions with unique or endemic climatic conditions in this geographical range. However, the most preferred thermal zone chosen by midges, and the optimal physiological range of 25–30 °C (77–86 °F), can be found between July and August throughout the California coast, the plateau regions, most of the north-central US, the central plains, and parts of the northeast [67,68]. In addition, this temperature range is also predominant in the Chihuahuan Desert and most of the southeast between May to September [67,68]. At the same time, fine-scale differences between the temperatures of surfaces and the shade relative to the surrounding air may create microclimates with optimal temperatures [69], allowing midges to behaviorally thermoregulate for extended portions of the year in any given location. Therefore, during VSV outbreaks, which often start in May in the southern states and July–August in the more northern states, infected midges will have an intermediate physiological performance (reproduction and survival) accompanied by a higher likelihood of having disseminated infections by the time they feed on a subsequent meal after infection.

Assuming that a field population of 3000 *C. sonorensis* midges (80% being females) [48] choose to rest in microclimates with temperatures ranging 25–30 °C after feeding on a VSV-infected host, by extrapolating our results, 1584 females (66%) would be able to complete two-to-three gonotrophic cycles, lasting an average of three days, resulting in three-to-four blood-feeding opportunities within 10 days. Moreover, this intermediate temperature range would also provide the optimal opportunity to maximize the infection processes involved in transmission. With infection rates above 80% and dissemination rates ranging from 30 to 48%, in this scenario, approximately 475–760 females would potentially survive the EIP and inflict infectious bites on susceptible hosts. By preferentially resting in areas closer in temperature to their ideal physiological range, VSV-infected midges may maximize their fitness-related traits along with providing highly permissive temperatures for VSV replication.

In an epidemiological context of vector species and environmental aspects, we have shown that *Culicoides* thermal behavior can have significant epidemiological implications on vector capacity and VSV transmission potential. However, the emergence of *Culicoides*-borne viruses worldwide indicates that pathogen–vector–host interactions are highly dynamic [70,71]. The rising average global temperature, along with more frequent heatwaves, large storms, and remarkably sunny and cloudy days, could have significant consequences for ecosystem stability [72,73,74]. Therefore, further work integrating additional relevant environmental conditions is necessary to investigate whether seasonal and daily fluctuating temperatures may significantly impact *Culicoides* vectorial capacity and thermal tolerance to temperatures outside their ideal physiological range.

## Data Availability

Raw data for figures available through Ag Data Commons within 30 months of publication.

## Supplementary Materials

The following supporting information can be downloaded at: https://www.mdpi.com/article/10.3390/insects13040372/s1, Table S1: Statistical results of Tukey’s multiple comparisons test to determine the differences in the gonotrophic cycle length, Table S2: Statistical results of the Mantel–Cox model applied to determine the survival probability, Table S3: *Culicoides* resting temperature preference after engorgement.
