# Supplementary material for: Effect of Constant Temperatures on Culicoides sonorensis Midge Physiology and Vesicular Stomatitis Virus Infection
Source: Insects. 2022 Apr 9;13(4):372. doi: 10.3390/insects13040372 (PMC9024736; doi:10.3390/insects13040372)
Supplement: Supplementary file 1 [file insects-13-00372-s001.zip › insects-1644477-supplementary.pdf]

Table S1. Statistical results of Tukey's multiple comparisons test to determine the differences in the gonotrophic cycle length

Gonotrophic Cycle	Comparison [1]	Adjusted *p-value*	*p-value* Summary [2]
1GC	20 vs. 25 °C	<0.0001	****
	20 vs. 30 °C	<0.0001	****
	20 vs. 35 °C	<0.0001	****
	25 vs. 30 °C	<0.0001	****
	25 vs. 35 °C	<0.0001	****
	30 vs. 35 °C	>0.9999	ns
2GC	20 vs. 25°C	<0.0001	****
	20 vs. 30 °C	<0.0001	****
	20 vs. 35 °C	<0.0001	****
	25 vs. 30 °C	<0.0001	****
	25 vs. 35 °C	<0.0001	****
	30 vs. 35 °C	>0.9999	ns
3GC	30 vs. 35 °C	>0.9999	ns

[1] Length of each GC was affected by temperature but not infection status. Results for VSV and Control groups are shown together for each temperature comparison. [2] $p > 0.05$, ns, not significant; **** $p \leq 0.0001$. Results shown in Figure 2a.

Table S2. Statistical results of the Mantel–Cox model applied to determine the survival probability

Comparison	χ^2	df	*p-value*	*p-value* Summary [1]
All groups	114.5	7	<0.0001	****
20 °C (VSV vs. Ctrl)	0.048	1	0.826	ns
25 °C (VSV vs. Ctrl)	0.003	1	0.957	ns
30 °C (VSV vs. Ctrl)	0.015	1	0.903	ns
35 °C (VSV vs. Ctrl)	0.005	1	0.946	ns

[1] $p > 0.05$, ns, not significant; **** $p \leq 0.001$. Results shown in Figure 2b.

Table S3. *Culicoides* resting temperature preference after engorgement

Blood Meal	Temperature (Infection Group)	Temperature Range Selected [2]					
		11 ± 1 °C	15–20 °C	20–25 °C	25–30 °C	30–35 °C	34 ± 1 °C
1BM	All [1]	2.6 ± 1.9	5.7 ± 2.4	31.4 ± 6.1	43.7 ± 6	12 ± 6.1	4.6 ± 3.4
2BM	20°C (VSV)	0	5 ± 2	24 ± 3.3	36 ± 8.6	18 ± 8.3	17 ± 3.8
	20°C (Ctrl)	3 ± 2	4 ± 3.3	17 ± 3.8	38 ± 7	21 ± 5	17 ± 6
	25°C (VSV)	2 ± 2.3	8 ± 6.5	28 ± 8.6	31 ± 6.8	18 ± 10.6	13 ± 6.8
	25°C (Ctrl)	3 ± 3.8	8 ± 7.3	19 ± 9.5	40 ± 5.7	24 ± 5.7	6 ± 9
	30°C (VSV)	1 ± 2	8 ± 4.6	24 ± 6.5	41 ± 6	15 ± 10	11 ± 3.8
	30°C (Ctrl)	3 ± 2	10 ± 4	28 ± 12.6	31 ± 5	15 ± 9.4	13 ± 11.5
	35°C (VSV)	1 ± 2	13 ± 13.2	34 ± 8	28 ± 11.8	14 ± 12.4	10 ± 5.1
	35°C (Ctrl)	1 ± 2	7 ± 6.8	20 ± 10	37 ± 5	27 ± 11.5	8 ± 8.6
3BM	20°C (VSV)	0	10 ± 2.3	31 ± 3.8	32 ± 5.7	19 ± 6	8 ± 3.3
	20°C (Ctrl)	2 ± 4	7 ± 5	27 ± 6	36 ± 3.3	19 ± 11	9 ± 2
	25°C (VSV)	3 ± 3.8	8 ± 6.5	32 ± 11.8	36 ± 5.7	11 ± 5	10 ± 5.2
	25°C (Ctrl)	3 ± 3.8	9 ± 6	26 ± 4	30 ± 6.9	24 ± 8.6	8 ± 3.3
	30°C (VSV)	3 ± 3.8	3 ± 2	29 ± 7.6	46 ± 15.5	14 ± 10.6	5 ± 3.8
	30°C (Ctrl)	1 ± 2	8 ± 0	32 ± 6.5	36 ± 10.8	21 ± 11	2 ± 2.3
	35°C (VSV)	6 ± 7.7	3 ± 6	30 ± 12	28 ± 3.3	19 ± 9.4	14 ± 7.7
	35°C (Ctrl)	1 ± 2	11 ± 5	28 ± 10.3	37 ± 13.2	14 ± 9.5	9 ± 8.9

[1] Data of both treatments were pooled to evaluate the resting temperature preference after the engorgement of midges with their first blood meal. [2] Percentage of midges (mean ± standard deviation) choosing to rest in each temperature zone.
